# Can a passive unilateral hip exosuit diminish walking asymmetry? A randomized trial

**DOI:** 10.1186/s12984-023-01212-w

**Published:** 2023-07-12

**Authors:** Kayla Kowalczyk, Mukul Mukherjee, Philippe Malcolm

**Affiliations:** 1 Department of Biomechanics and Center for Research in Human Movement Variability, University of Nebraska at Omaha, 6160 University Drive, Omaha, NE 68182-0860 USA; 2 UGA Concussion Research Laboratory, Department of Kinesiology, University of Georgia, Athens, GA USA

**Keywords:** Split-belt, Exoskeleton, Adaptation, Walking, Biomechanics

## Abstract

**Background:**

Asymmetric walking gait impairs activities of daily living in neurological patient populations, increases their fall risk, and leads to comorbidities. Accessible, long-term rehabilitation methods are needed to help neurological patients restore symmetrical walking patterns. This study aimed to determine if a passive unilateral hip exosuit can modify an induced asymmetric walking gait pattern. We hypothesized that a passive hip exosuit would diminish initial- and post-split-belt treadmill walking after-effects in healthy young adults.

**Methods:**

We divided 15 healthy young adults evenly between three experimental groups that each completed a baseline trial, an adaptation period with different interventions for each group, and a post-adaptation trial. To isolate the contribution of the exosuit we compared a group adapting to the exosuit and split-belt treadmill (Exo-Sb) to groups adapting to exosuit-only (Exo-only) and split-belt only (Sb-only) conditions. The independent variables step length, stance time, and swing time symmetry were analyzed across five timepoints (baseline, early- and late adaptation, and early- and late post-adaptation) using a 3 × 5 mixed ANOVA.

**Results:**

We found significant interaction and time effects on step length, stance time and swing time symmetry. Sb-only produced increased step length asymmetry at early adaptation compared to baseline (p < 0.0001) and an after-effect with increased asymmetry at early post-adaptation compared to baseline (p < 0.0001). Exo-only increased step length asymmetry (in the opposite direction as Sb-only) at early adaptation compared to baseline (p = 0.0392) but did not influence the participants sufficiently to result in a post-effect. Exo-Sb produced similar changes in step length asymmetry in the same direction as Sb-only (p = 0.0014). However, in contrast to Sb-only there was no significant after-effect between early post-adaptation and baseline (p = 0.0885).

**Conclusion:**

The passive exosuit successfully diminished asymmetrical step length after-effects induced by the split-belt treadmill in Exo-Sb. These results support the passive exosuit’s ability to alter walking gait patterns.

**Supplementary Information:**

The online version contains supplementary material available at 10.1186/s12984-023-01212-w.

## Background

Over 8 million people in the United States live with lingering symptoms following stroke [1]. This disorder alters the functioning of the central nervous system (CNS), leading to impaired motor control (i.e., hemiparesis or partial paralysis) and the possibility of asymmetric walking patterns [2–6]. The ability to walk enables individuals to perform different home- or community-based activities and maintain healthy, active lifestyles. CNS motor and sensory pathways produce the framework for lower extremity muscles and joints to work in unison to move the body forward [7, 8]. Disruptions to neurological function can alter the symmetrical movement of the lower extremity joints and can sometimes lead to more inefficient asymmetric patterns. Interlimb asymmetry can take on temporal (e.g., stance or swing time) and/or spatial (e.g., step length) forms [2, 9, 10]. Consequently, reductions in preferred walking velocity and lower extremity range of motion may result from altered step length and modified stance duration (the degree of each varies on an individual level) [2–4, 9]. Altered mechanics limit mobility and increase effort, energy costs, and the risk of falls during ambulation in affected populations [5, 11–14]. This hemiparetic interference with daily activities may deteriorate overall health, which can lead to an increased risk for future medical issues in patient populations.

The utilization of novel perturbations to alter walking gait symmetry has produced short-term ambulation improvements. By utilizing these perturbations, the CNS can be trained to adapt to complex, unexplored environments through the integration of sensory feedback during ongoing movement [15–17]. To influence the asymmetric walking gait of patients following stroke, previous work explored perturbing ambulation through weighting the less-paretic limb, which is also known as constraint-induced movement therapy (CIMT) [18, 19]. After completing a 20-minute treadmill walking session with a weight attached to the less-paretic limb, participants increased their gait speed and step length from baseline to the follow-up [18]. This finding suggests a short-term walking gait improvement as a result of less paretic limb weighting. Long-term investigations of multiple CIMT training sessions found that participants developed improvements in stride length after completing treadmill walking with additional weight on their less-paretic limb [19]. Despite the improvements obtained using less-paretic limb weighting training, researchers found no significant differences compared to controls that completed treadmill walking training alone. The results suggest that treadmill training alone sufficiently improved walking ability. Additionally, adding weight at the ankle increases metabolic demands and destabilizes walking gait, which creates adverse issues for populations experiencing increased metabolic demands from abnormal gait [13, 20–22].

During walking each limb adapts independently to the environment, allowing for leg-specific responses to perturbations [23]. This concept is especially relevant during the use of a split-belt treadmill, a treadmill with separate belts for the left and right leg that can move at different velocities. Split-belt training has been used to perturb the walking environment of stroke patients to assess their ability to adapt to new locomotion patterns [24]. For example, participants following stroke altered their step length and stance times to accommodate different belt velocities on a split-belt treadmill [24]. The participants with asymmetries at baseline developed symmetrical step-length after-effects (adaptations to the perturbation) once the belts returned to a tied condition. This suggests that a damaged CNS does not restrict, short-term symmetrical walking adaptations [24]. Long-term investigations of the effects of split-belt walking in stroke populations found improved step length asymmetry compared to baseline initially after completing the protocol. However, the participants did not maintain improvements one and three months after the intervention [25]. Temporal walking symmetry improvements (i.e., stance or double support time) remained unchanged across all collection time points [25]. Although these studies provided the framework for short-term gait adaptations, split-belt training for long-term retention and rehabilitation is not particularly convenient (e.g., at home training interventions). The need to develop accessible rehabilitation techniques for patient populations is sizable and critical. One avenue that may improve access to long-term walking gait therapies involves the application of external wearable devices, such as exoskeletons (or exosuits).

Previously, researchers have used exoskeletons to manipulate spatiotemporal, kinematic, and kinetic movement characteristics. Robotic (active) exoskeletons use software and powered actuation systems to apply forces at specific times during a movement pattern, such as walking gait [26–29]. Newer designs significantly reduced the size of the devices and power actuation sources and improved the comfort of active exoskeletons [30, 31]. Passive exoskeletons consist of elastic elements, such as springs or mechanically triggered clutches that deform and return stored elastic energy at a different point during the movement [32, 33]. Unlike active exoskeletons, passive devices require no external power to apply resistance or assistance [34]. The simplicity of a passive elastic exoskeleton allows the individual operator to put them on in a few minutes, dramatically reduces the cost of materials, and permits device application outside of research or clinical rehabilitation settings [35–37]. Many exoskeleton designs focus on assisting the ankle. In the case of impaired patient populations, a hip device may provide further benefit because adding weight at the hip is less destabilizing and metabolically less expensive during locomotion compared to adding weight at the ankle [21, 22]. Furthermore, the hip joint plays a critical role in efficient limb advancement throughout walking by providing approximately 40–50% of the positive power required for forward progression during healthy gait [38–40]. From a musculotendon perspective, the hip extensors and flexors function as springs that store elastic energy during one phase of walking and impart the stored energy in another phase. Specifically, hip extensors (e.g., hamstrings, gluteus maximus) assist with the deceleration of the thigh during the swing phase of walking and accelerating at the beginning of stance; these muscles help stabilize the body to lower extremity forces [38, 41, 42]. The hip flexors (e.g., rectus femoris, iliopsoas, sartorius) actively progress the thigh forward during the swing phase and passively aid leg deceleration during the second half of stance [41, 43]. Due to the importance of the hip for walking, using a passive exoskeleton or exosuit to perturb the hip motion by adding a force that is not naturally produced by the body offers a promising avenue to induce adaptative changes.

Typically, wearable devices, such as exoskeletons, are used to provide assistance. However, they may also yield resistance to promote adaptations similar to those observed with split-belt perturbations. Recent studies have explored the use of exoskeletons [29, 44] and customized perturbation footwear [45] to achieve such adaptation effects. Two notable studies examined the effects of a powered unilateral ankle exosuit and a powered unilateral hip exoskeleton on healthy participants, with the goal of uncovering benefits that could ultimately be useful for post-stroke therapy [29, 44]. Both studies observed temporary increases in range of motion (plantarflexion in the ankle exosuit study and hip motion in the hip exoskeleton study), but neither reported significant step-length adaptation effects. The hip exoskeleton study highlighted common challenges in fitting rigid exoskeletons to the complex hip joint motion, supporting the idea of conducting similar research using a passive, soft hip exosuit.

The specific objective of our study was to determine if a passive unilateral hip exosuit can diminish asymmetric walking gait patterns in healthy participants. In order to induce walking asymmetry in healthy participants, we used a split-belt treadmill. Previous studies found that the split-belt paradigm leads to asymmetrical walking patterns in healthy young adults when initially introduced and asymmetrical after-effects upon return to a tied configuration [46–49]. We hypothesized that wearing the exosuit would reduce split-belt treadmill induced asymmetrical step length, stance time, and swing time after-effects in healthy individuals. This study’s findings could establish the proof-of-concept required for future research in neurologically afflicted patient populations and the foundation for an accessible community-based, long-term rehabilitation strategy to assist patients in their recovery.

## Methods

### Participants

Fifteen healthy young adults (6 females, 9 males; 13 right-footed, 2 left-footed; age = 24.13 ± 2.47 years; mass = 72.2 ± 11.9 kg; height = 172.5 ± 9.8 cm; mean ± sd.) (Additional File 6-Table 10) from a convenience sample (recruited from the University of Nebraska at Omaha campus) volunteered to participate in this study. Exclusion criteria included individuals with a history of neurological (e.g., stroke and multiple sclerosis), cardiovascular (e.g., heart arrhythmias or emphysema), or musculoskeletal (e.g., myopathy or arthritis) disorders, and having had of lower extremity surgery within the previous two years. We screened for these conditions using a health-history questionnaire that prospective participants completed before the scheduled data collection period. Leg dominance was determined using the validated question, “what leg would you use to kick a ball,“ from a study conducted by Melick et al. [50]. To be included in the study, participants must have been between the ages of 19 and 40 with the capacity to walk without assistance. The sample size was chosen based on typical sample sizes of similar studies [24, 51, 52]. We did not determine the sample size based on a statistical power analysis since we did not have prior knowledge of the effect size. We did not adjust the sample size during the trial based on an interim analysis. The study was approved by the University of Nebraska Medical Center Institutional Review board and all participants signed the approved consent form before participating.

### Experimental protocol

**Fig. 1:**
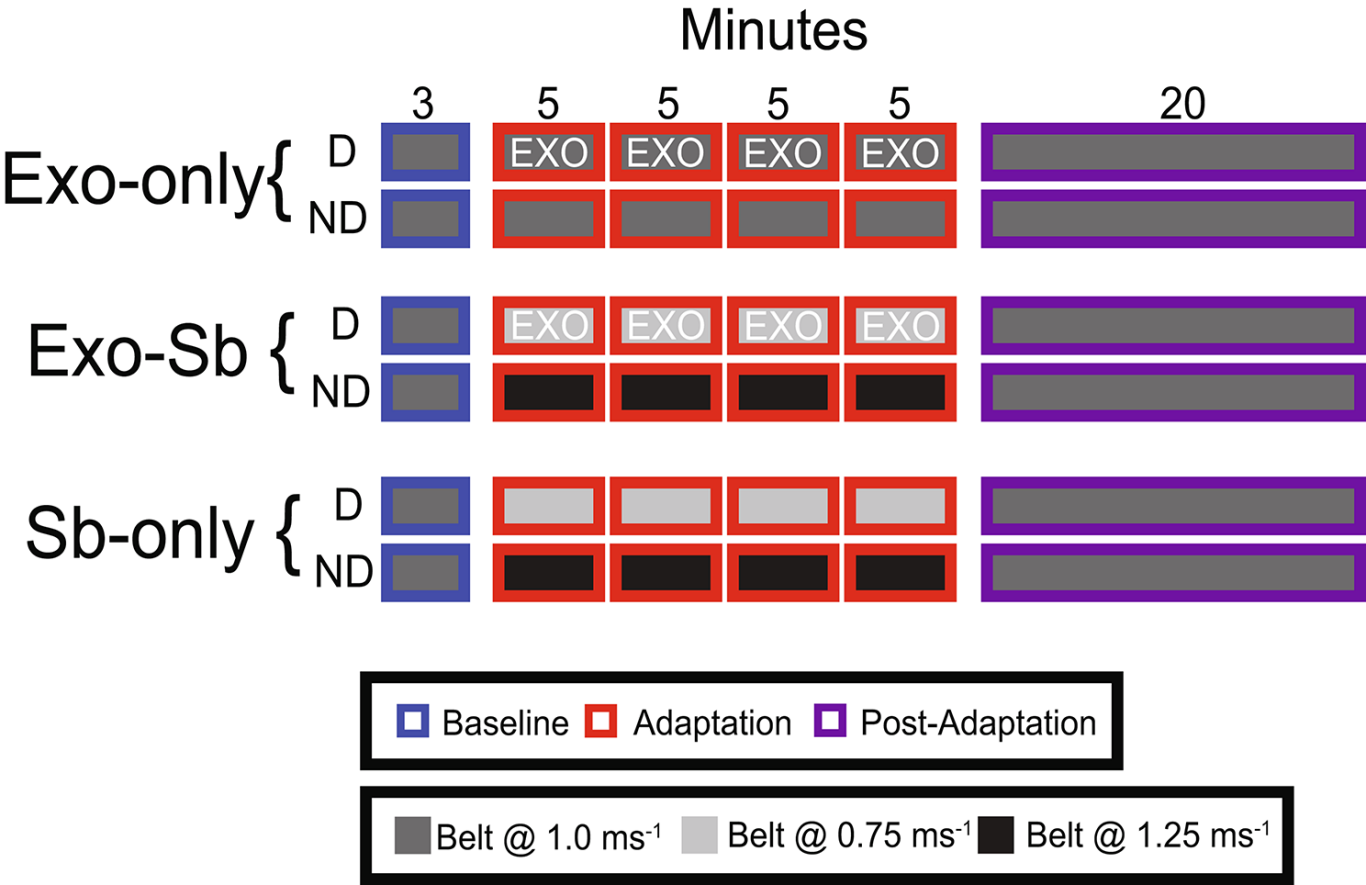
Protocol Set-up. Times, belt speeds, and trials for each experimental group. The time periods, Baseline (blue), Adaptation (red), and Post-adaptation (purple), are represented by the outlines. Numbers at the top represent the minutes spent in each section. D stands for dominant and ND stands for non-dominant. The color filling represents the individual belt velocities of the split-belt treadmill. Grey represents the standard velocity at 1.0 ms^− 1^, light grey represents the slow velocity at 0.75 ms^− 1^, and black represents the fast velocity at 1.25 ms^− 1^. The exosuit resists the leg on the slow belt in Exo-Sb

Data collection took place in the virtual reality lab in the Biomechanics Research building at the University of Nebraska. We allocated participants evenly into three parallel groups with five participants each: Exosuit-only (Exo-only), Split-belt only (Sb-only), and Simultaneous (Exo-Sb). All participants in each group walked on an instrumented Split-belt Treadmill (Bertec®, Columbus, Ohio) that sampled kinetic data at 1000 Hz. Each group completed a 3-minute baseline walking trial with the belts tied at 1.0 ms^− 1^. Following the baseline trial, participants began the adaptation trials. Exo-only completed four 5-minute trials while wearing a unilateral passive hip exosuit on their dominant limb with the belts tied at 1.0 ms^− 1^. Sb-only completed four 5-minute trials with the belts of the treadmill at different velocities such that participants walked with their dominant limb on the slow belt at 0.75 ms^− 1^ and with their non-dominant limb on the fast belt at 1.25 ms^− 1^. Exo-Sb followed the same protocol as Sb-only for the adaptation trials with the addition of a unilateral passive hip exosuit on the dominant limb. After completing the fourth adaptation trial, all three groups began a post-adaptation trial lasting 20-minutes with the belts tied at 1.0 ms^− 1^. Figure 1 provides a detailed image of the experimental protocol.

We measured kinematics at 100 Hz using a calibrated 16-camera motion capture system (Vicon Motion Systems, Ltd, Oxford, United Kingdom). Participants wore a close-fitting singlet and their preferred walking shoes throughout the entirety of the collection. We placed a marker set consisting of 33 retro-reflective markers (12.7 mm) on bony landmarks bilaterally on the feet (head of 1st and 5th metatarsal, base of the 2nd phalange, calcaneus, heel), shank (medial and lateral malleoli, tibial tuberosity, lateral shank), knee (medial and lateral epicondyles of the femur), thigh (lateral thigh, greater trochanter, ventral-distal thigh), and pelvis (anterior superior iliac spine, posterior superior iliac spine, sacrum) to track lower extremity kinematics following a modified Helen-Hayes marker set. We put the retro-reflective markers directly on the skin, on the surface of the singlet, or on top of the shoes over the foot landmarks. Participants wore a safety harness attached to a ceiling mount throughout all collection trials. The harness did not support body weight.

### Exosuit design

**Fig. 2:**
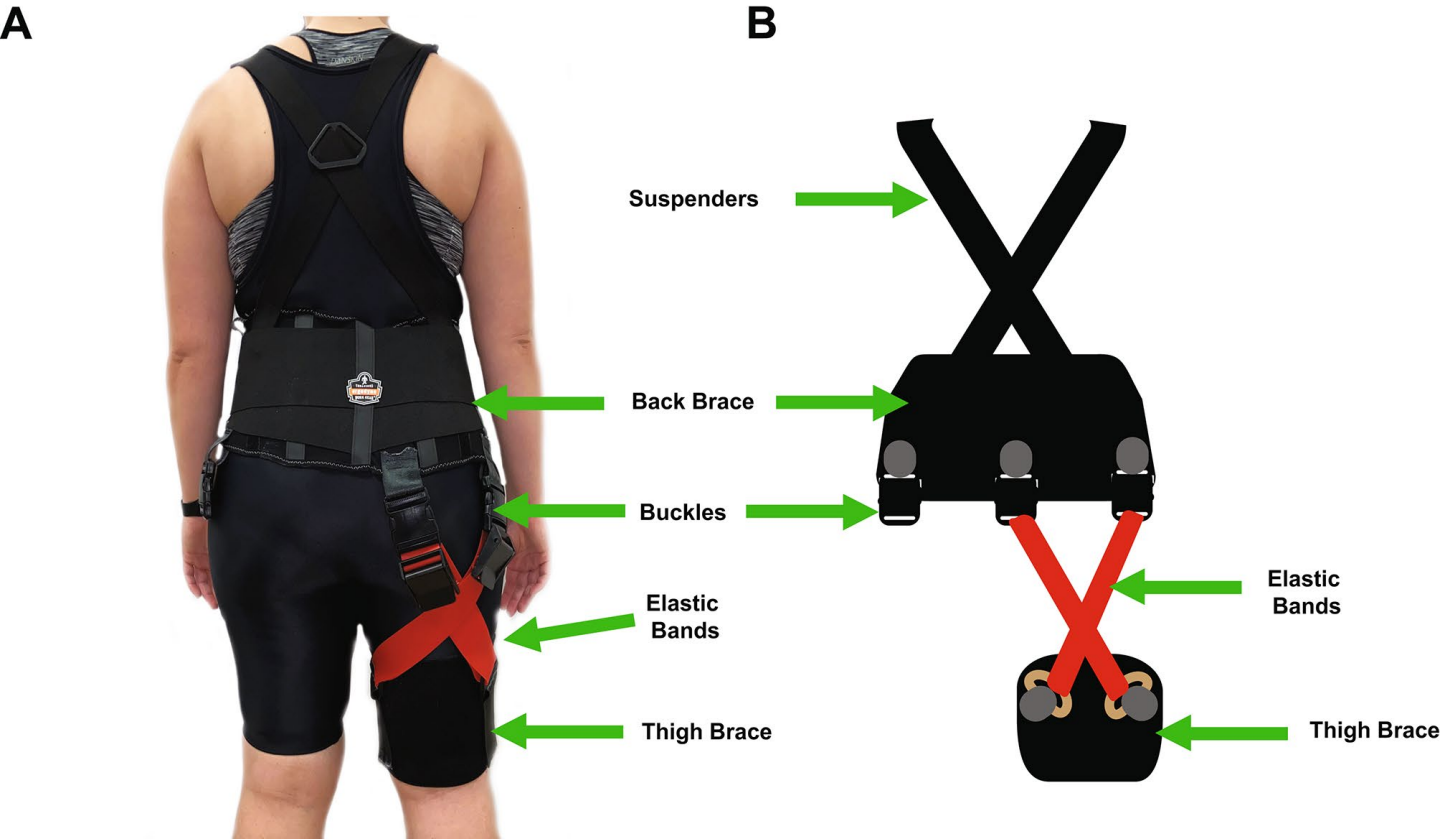
Passive Hip Exosuit Design. (**A**) A photograph of the passive hip exosuit on a participant. (**B**) A diagram of the device with band attachment locations

We assembled the passive unilateral exosuit based on previous designs [4, 36]. Figure 2 shows the passive exosuit built for this study. The proximal portion consists of a Proflex® back brace (Ergodyne, Saint Paul, Minnesota, United States) with suspender straps. We sewed buckles onto the back brace to switch the passive portion based on leg dominance and for easy attachment and removal of the bands during the data collection protocol. A soft thigh brace composed the distal portion of the device with D-rings sewn onto the sides. We utilized Fit Simplify© heavy bands as the passive elastic component of the exosuit. The device had bands positioned posteriorly in a crisscross fashion to assist hip extension and resist hip flexion.

### Elastic band stiffness test

Similar to the methods of Panizzolo et al., we implemented a tensile test using an Instron Materials Testing Machine (Instron®, Norwood, Massachusetts) to quantify the exosuit’s elastic band stiffness [53]. The machine elongated the band at 1 mms^− 1^. Upon completion, we generated a graph of the force-elongation relationship, which can be found in the additional files (Additional File 1). After applying a linear line of best fit to the graph, we employed the following equation to describe the relationship between force and elongation:

1 $$F= {K}_{el}\cdot E+Y\_Intercept$$

*F* represents the force, *K*_*el*_ represents the stiffness coefficient, and *E* signifies elongation. Based on this equation, we determined the stiffness coefficient as 0.60 Nmm^− 1^.

To estimate the force contribution of the bands during the experimental trials, we placed retro-reflective markers 50 mm apart on a slack band for each participant in Exo-only and Exo-Sb. We calculated the distance between the markers during the trials by employing a custom MATLAB (MathWorks Inc., Natick, Massachusetts) script. The slack marker distance (50 mm) was then subtracted from the experimental distances to calculate the elongation of the band. We used the following equation to estimate the force [54]:

2 $$F= {K}_{el}\cdot E$$

We extracted the stored elastic force at the heel strike of the first ten strides of the first adaptation trial for seven participants among Exo-only and Exo-Sb (three participants had too much missing data from the band markers). We multiplied the elongation value with the coefficient of stiffness to calculate the amassed force at heel strike. Based on this, we determined that each band applies about 6.9 ± 1.4 N of elastic force at heel strike.

### Data analysis and processing

We completed initial kinematic and kinetic data processing in Visual 3D (C-Motion Inc., Germantown, MD). Data was filtered in Visual 3D using a fourth-order low-pass Butterworth filter with a 6 Hz cutoff frequency. We exported ground reaction forces (GRF) and heel marker locations to MATLAB. Using a custom script, we used GRF data to detect the left and right heel contact and toe-off times of each trial. We subtracted the initial heel contact time from the next heel contact time on the same side for both the left and right sides to calculate stride time. Our primary outcome was step length symmetry index, and our secondary outcomes were stance time and swing time symmetry. We calculated step length as the anterior-posterior distance between the leading leg heel marker and the trailing leg heel marker at heel contact of the leading leg in accordance with previous studies [24, 47, 55, 56]. To determine stance time, we calculated the time between heel contact and toe-off on the same side, and for swing time, we calculated the time between toe-off and heel contact on the same side. To assess asymmetries between limbs, we used the following symmetry index (SI) equation similar to Robinson et al. and others [47, 56–59]:

3 $$SI= \frac{F - S}{F + S}\cdot 100$$

where F stands for the leg on the fast belt or the leg that was not resisted by the exosuit, and S stands for the leg on the slow belt or leg that was resisted by the exosuit brace. Values of zero represent complete symmetry. Larger negative or positive values represent asymmetries between legs.

### Statistical analysis

For our statistical analysis, we compared the average step length, stance, and swing time SI values at five different time points: baseline (BL), early adaptation (EA), late adaptation (LA), early post-adaptation (EP), and late post-adaptation (LP). BL, LA, and LP correspond to the average SI values during the last ten strides of baseline, the final adaptation trial, and the tenth minute of post-adaptation, respectively. EA and EP refer to the first ten strides of the adaptation and post-adaptation periods. We checked the normality of all dependent variables using a Shapiro-Wilk test. Data was considered as an outlier if the value was more than two standard deviations away from the group mean. To assess SI differences between and within groups at the specified time points, we implemented a 3 × 5 mixed ANOVA. Significant between-group interactions underwent follow-up simple effects tests using one-way ANOVAs. We used follow-up one-way repeated measures ANOVAs and paired comparisons to determine specific within-group time point differences. We set the significance threshold at p < 0.05 with a Bonferroni correction for simple comparison tests. The researchers and the participants were not blinded for this initial study.

## Results

### Demographics and outlier verification

We found no differences in height (F = 0.026, p = 0.974) and weight (F = 0.290, p = 0.753) among the three experimental groups. One participant from Exo-Sb had a step length symmetry index at EP over two standard deviations from the group mean. We considered this participant an outlier and removed him from the step-length SI analysis. There were no other outliers. We report Huynh-Feldt corrected p-values for models that did not pass Mauchly’s Test of Sphericity.

### Temporal gait asymmetry is unaffected by the unilateral hip exosuit

For stance time SI, we found a significant interaction between group and time with a large effect size (F = 9.47, p-corrected = 0.00014, η^2^ = 0.521). Follow-up tests, determined differences occurred at EA (F = 13.8, p = 0.0039, η^2^ = 0.697) and LA (F = 13.1, p = 0.0048, η^2^ = 0.686; Additional File 6-Table 1). No differences between groups were found at BL or either post-adaptation timepoint. At EA and LA, Exo-Sb (EA: p = 0.001; LA: p = 0.001) and Sb-only (EA: p = 0.0052; LA: p = 0.0113) had larger negative stance time SI compared to Exo-only.

**Fig. 3:**
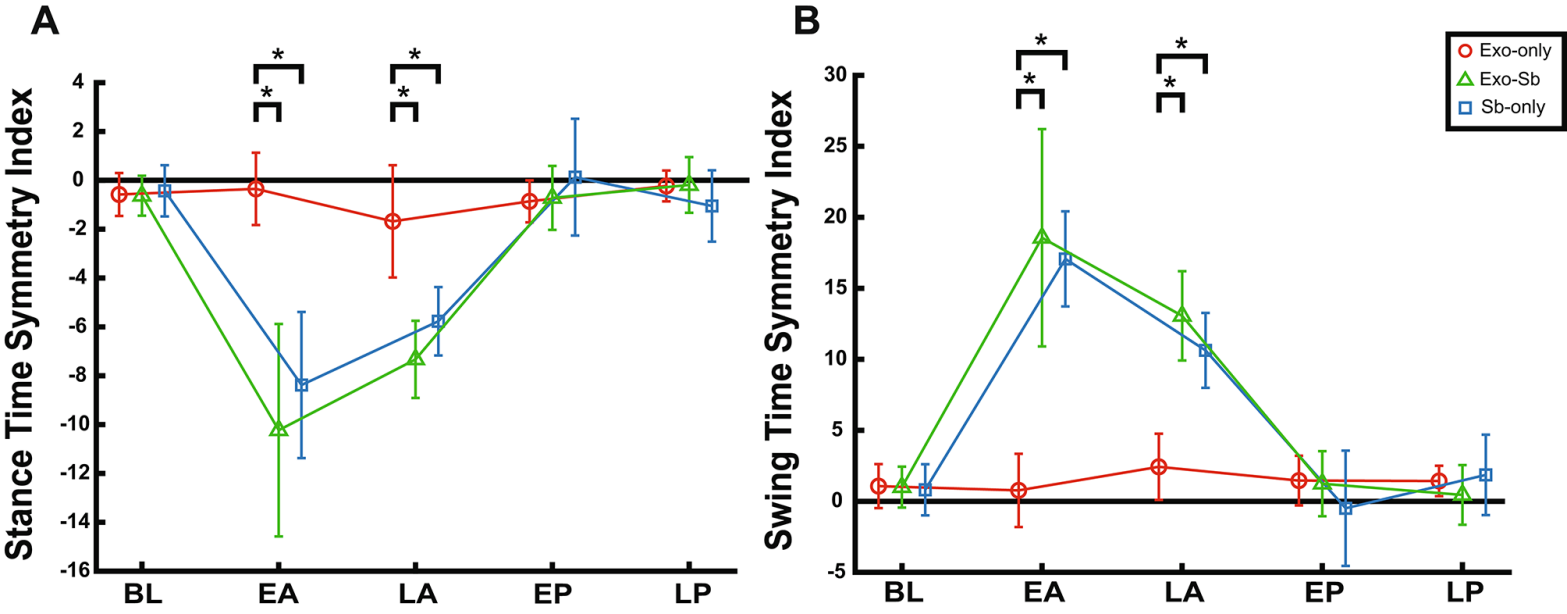
The Passive Exosuit Does Not Alter Induced Temporal Asymmetry. Panel A displays Exo-only (red), Exo-Sb (green), and Sb-only (blue) stance time SI values and Panel B displays swing time SI values for the same groups at baseline (BL), early adaptation (EA), late adaptation (LA), early post-adaptation (EP), and late post-adaptation (LP). The “*” marks significant differences between groups at specific timepoints

Similar findings were seen for the swing time SI (Fig. 3). We found a significant interaction between group and time with a large effect size for swing time SI (F = 13.59, p < 0.0001, η^2^ = 0.602). Follow-up tests confirmed differences occurred at EA (F = 19.1, p = 0.00093, η^2^ = 0.761) and LA (F = 20.9, p = 0.00062, η^2^ = 0.777). No differences between groups were found at BL or either post-adaptation timepoint (Additional File 6-Table 2). At EA and LA, Exo-Sb (EA: p = 0.0004; LA: p = 0.001) and Sb-only (EA: p = 0.0008; LA: p = 0.0014) had significantly larger positive swing time SI values compared to Exo-only (Fig. 3).

Follow-up tests were run on all three groups for stance and swing time SI. We discovered no differences between timepoints for Exo-only (F = 01.22, p-corrected = 0.3412, η^2^ = 0.151). Within group stance time SI differences were significant for Exo-Sb (F = 28.09, p = 0.0017, η^2^ = 0.812) and Sb-only (F = 17.08, p < 0.0001, η^2^ = 0.781). Follow-up pairwise comparisons determined that Sb-only and Exo-Sb had significantly larger negative stance time SI values at EA and LA than BL (Additional File 6-Tables 3 and 4, and Additional File 2). We discovered no differences between timepoints for Exo-only (F = 0.9042, p-corrected = 0.4847, η^2^ = 0.095). Within group swing time SI differences were significant for Exo-Sb (F = 29.65, p = 0.0021, η^2^ = 0.815) and Sb-only (F = 28.49, p < 0.0001, η^2^ = 0.862). Follow-up pairwise comparisons determined that Sb-only and Exo-Sb had significantly increased positive swing time SI values at EA and LA compared to BL (Additional File 6-Tables 5 and 6, and Additional File 3).

### Between group step length asymmetry differences persist at early adaptation and early post-adaptation

**Fig. 4:**
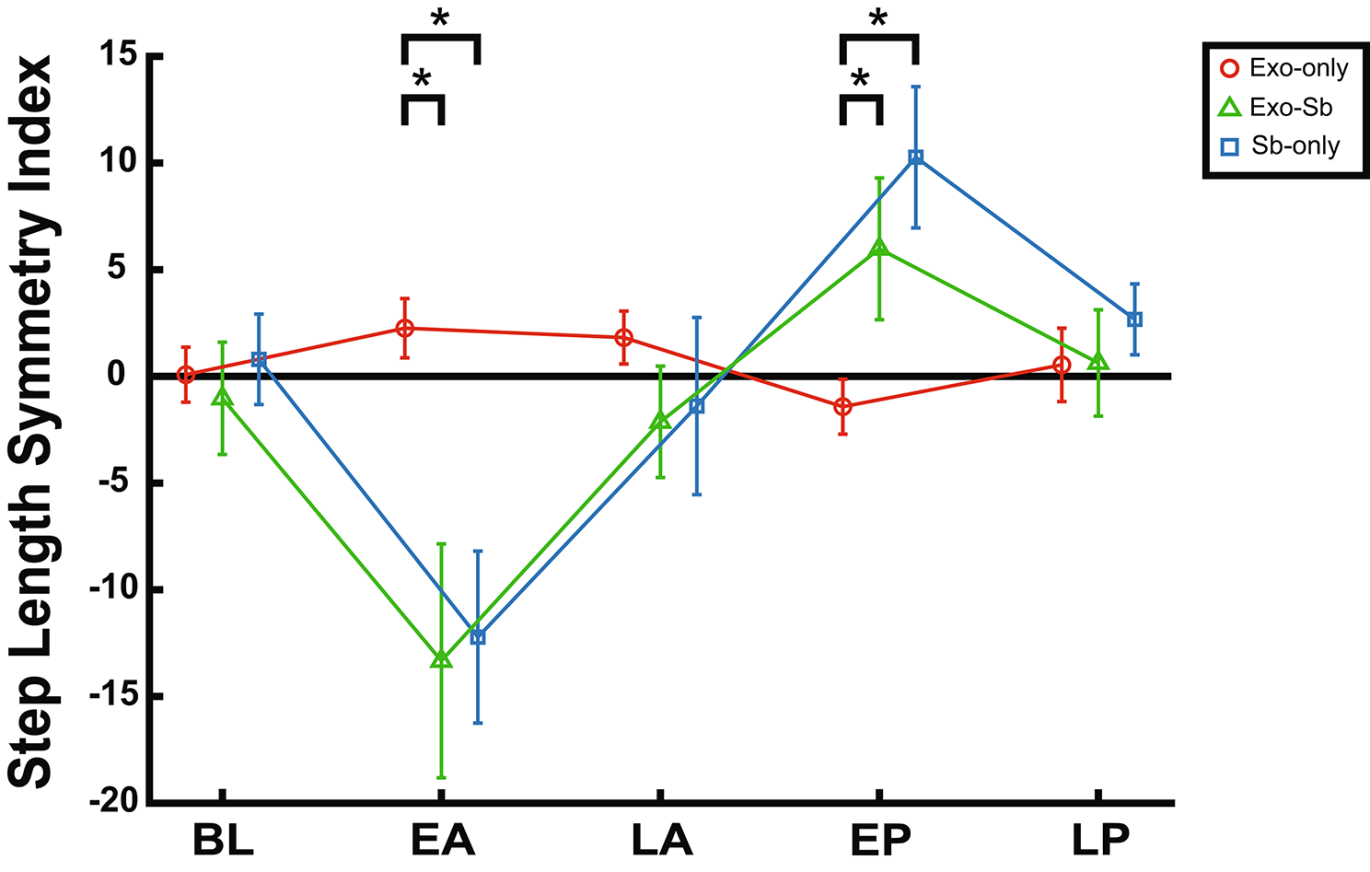
Between-Group Step Length SI Differences Persist. Step length SI values for Exo-only (red), Exo-Sb (green), and Sb-only (blue) at baseline (BL), early adaptation (EA), late adaptation (LA), early post-adaptation (EP), and late post-adaptation (LP). The “*” marks significant differences between groups at specific timepoints. Exo-only produced changes in the opposite direction as split-belt conditions which may explain the difference between Exo-Sb and Sb-only at EP

We found an interaction between group and time with a large effect size for step length SI (F = 29.55, p-corrected < 0.0001, η^2^ = 0.711). Follow-up tests determined differences between groups occurred at EA (F = 24.50, p = 0.00045, η^2^ = 0.816) and EP (F = 22.90, p = 0.0006, η^2^ = 0.806). No differences between groups were found at BL, LA, or LP (Table 7). At EA, Exo-Sb (p = 0.0003) and Sb-only (p = 0.0003) had significantly larger negative step length SI values compared to Exo-only. Exo-Sb (p = 0.0063) and Sb-only (p = 0.0001) both produced significantly larger positive step length asymmetry values at EP compared to Exo-only (Fig. 4). No step length SI differences between Exo-Sb and Sb-only were found at either time point (EA: p = 1; EP: p = 0.1215).

### A unilateral hip exosuit diminished within-group step length asymmetry post-effects

**Fig. 5:**
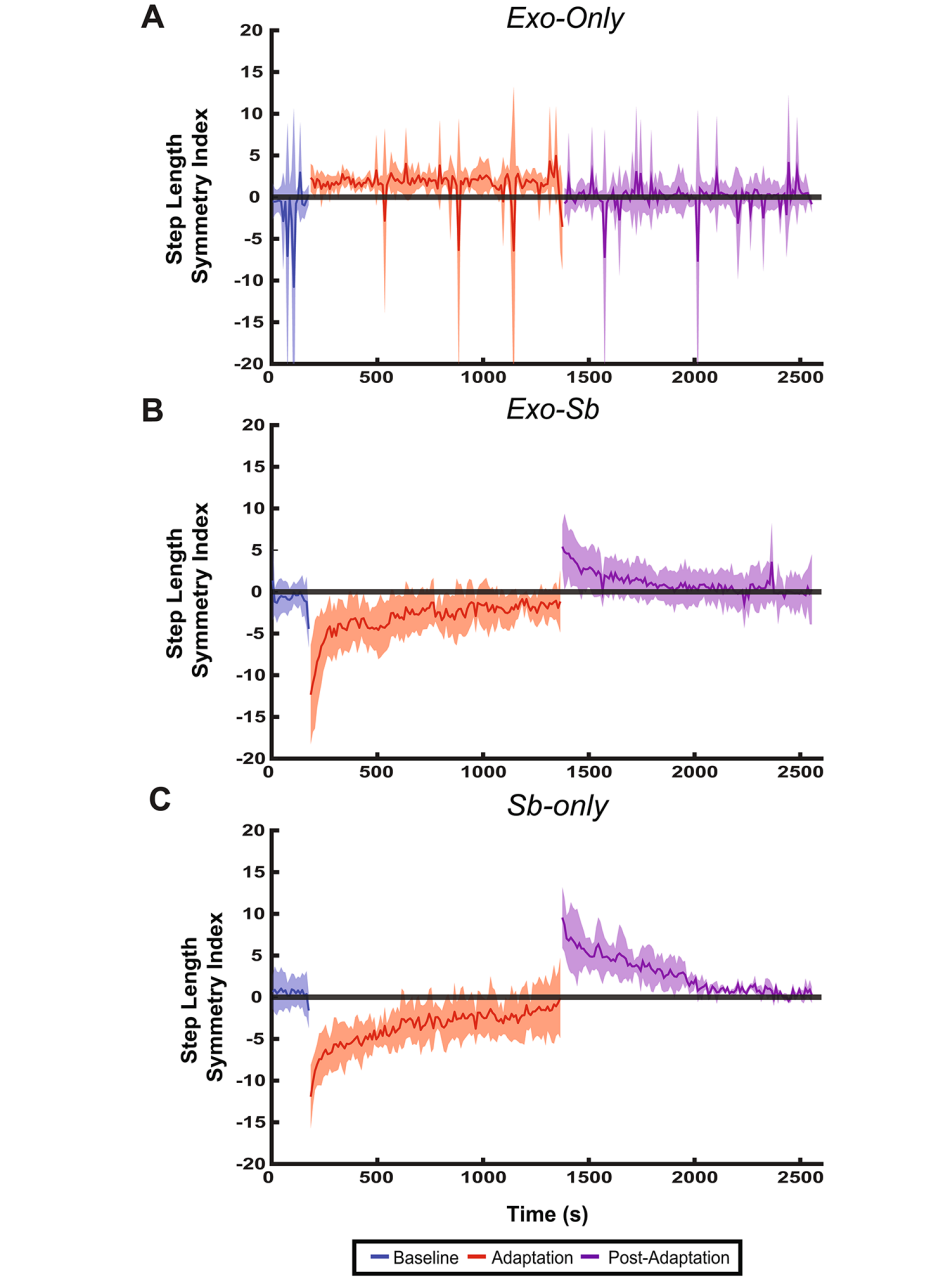
The Passive Hip Exosuit Diminishes Mean Step Length Asymmetry During Post-Adaptation. Panel **A**, **B**, and **C** display the average step length SI values and their standard deviation for Exo-only, Exo-Sb, and Sb-only respectively. The solid blue line represents the mean during baseline, the solid red line represents the mean during adaptation, and the solid purple line represents the mean during post-adaptation. The shaded areas in blue, red, and purple are the standard deviation at specific times during baseline, adaptation, and post-adaptation respectively. In contrast to Sb-only, Exo-Sb did not have a significant after-effect in step length asymmetry between EP (start of post-adaptation) and BL (end of baseline)

We ran one-way repeated measure ANOVAs on each group to assess the effect of individual perturbations on step length asymmetry (Table 1, Fig. 5). We only found differences across time points for Sb (F = 90.60, p < 0.0001, η^2^ = 0.865). Follow-up paired comparisons determined that participants in Sb-only had significantly lower negative step length SI values at EA compared to BL (p < 0.0001), LA (p < 0.0001), EP (p < 0.0001), and LP (p < 0.0001). At LA, Sb-only had significantly larger negative step length SI values compared to EP (p < 0.0001) and LP (p = 0.04). At EP, Sb-only produced significantly larger positive step length SI values compared to BL (p < 0.0001) and LP (p < 0.0001) (Additional File 6-Table 7).

**Table 1:** Step Length SI Group Means and SD Across Time Points

	Groups							
	*Exo-only*		*Sb-only*		*Exo-Sb*			
**Time**	**Mean**	**SD**	**Mean**	**SD**	**Mean**	**SD**	**P-value**	
*BL*	0.08	1.29	0.80	2.12	-1.03	2.63	P_Interaction_ < 0.001* P_Time_ < 0.001* P_Groups_ = 0.191	
*EA*	2.26	1.39	-12.22	4.03	-13.33	5.48		
*LA*	1.82	1.24	-1.39	4.15	-2.13	2.61		
*EP*	-1.42	1.29	10.27	3.31	5.98	3.32		
*LP*	0.54	1.72	2.67	1.66	0.63	2.49		

P-values from mixed-model ANOVA; BL: Baseline, EA: Early-adaptation, LA: Late-adaptation, EP: Early Post-adaptation, LP: Late Post-adaptation, Exo-only: Exosuit-only, Sb-only: Split-belt only, Ex-Sb: Combined Exosuit and Split-belt, SD: Standard Deviation

* Represents values < 0.05

Exo-only had differences in step length SI across timepoints (F = 10.20, p = 0.0003, η^2^ = 0.523) with a strong effect size. Follow-up paired comparisons determined that participants in Exo-only produced larger positive step length SI values at EA compared to BL (p = 0.0392) and EP (p = 0.0003). At EP, Exo-only had larger negative step length SI values compared to LA (p = 0.0013) but not to BL (p = 0.3489) (Additional File 6-Table 8).

Differences in step length SI values across time points with a large effect size were found for Exo-Sb (F = 19.84, p < 0.0001, η2 = 0.814). Follow-up paired comparisons determined that Exo-Sb produced larger negative step length SI values at EA compared to BL (p = 0.0014), LA (p = 0.0031), EP (p < 0.0001), and LP (p = 0.0004). At EP, Exo-Sb was found to have larger positive step length SI values compared to LA (p = 0.0357) but not to BL (p = 0.0885) (Additional File 6-Table 9, Additional File 4).

## Discussion

This study investigated how a passive unilateral hip exosuit modified the temporal and spatial gait characteristics of healthy individuals with induced walking asymmetry (i.e., split-belt treadmill walking). We hypothesized that wearing the passive unilateral hip exosuit while walking on a split-belt treadmill would diminish induced asymmetry from the split-belt during the initial adaptation trial and after the belts returned to a tied configuration. The experimental results from this study partially support our hypothesis.

In healthy individuals, we found that combining the exosuit with split-belt walking (i.e., Exo-Sb) did not change the step length asymmetry during EA compared to participants in Sb-only. Additionally, the results indicated that the passive hip exosuit did not mitigate any temporal (i.e., stance and swing time) asymmetries while walking on the split-belt treadmill. However, in support of our hypothesis, results showed that combining the exosuit with split-belt walking (Exo-Sb) diminished the within-group step length asymmetry after-effect when treadmill belts returned to the same velocity at the start of the post-adaptation period.

Previous studies in healthy participants found that temporal parameters, such as stance and swing time, changed immediately with the introduction of a split-belt perturbation [24, 48, 52]. The asymmetric stance and swing times persisted throughout split-belt walking and returned to their baseline values once the belts returned to a tied configuration [24, 48, 52]. The results from this current study match those of the previous studies. Participant stance and swing time asymmetry increased after introducing different belt speeds in both Exo-Sb and Sb-only conditions, with the passive device failing to diminish the imposed asymmetry in Exo-Sb. In both groups, temporal asymmetry persisted until the end of the adaptation trials compared to baseline, against our initial hypothesis. Additionally, stance and swing times for the healthy individuals in Sb-only and Exo-Sb returned to baseline symmetry values at EP when both belt velocities returned to a tied speed.

The initial change in velocity at EA resulted in larger negative step length asymmetries for participants in Sb-only and Exo-Sb conditions (i.e., the leg on the fast-belt or the non-dominant leg took shorter steps compared to the leg on the slow belt or the dominant leg), again similar to previous studies examining the split-belt paradigm [47, 56, 60]. As in the previous studies, participants in Sb-only and Exo-Sb then slowly returned to BL step length symmetry levels throughout the four adaptation trials [47, 56]. The initial step-length asymmetry adaptive change found in participants from Exo-Sb and Exo-only from BL to EA occurred in opposite directions. This indicates the split-belt perturbation overpowered the effect of the hip exosuit. Since the step length symmetry returned to BL levels by the end of the adaptation trials similarly in all groups, it seems that combining the exosuit with split-belt walking had no significant effect at both time points during the adaptation period.

The removal of each of the perturbations resulted in a significant within-group step length symmetry post-effect in participants from Sb-only, suggesting that the addition of the exosuit diminished within-group step length symmetry post-effects in participants from Exo-Sb. The removal of the exosuit led participants in Exo-only to walk with negative step length asymmetry values at EP (i.e., the leg that experienced the passive exosuit resistance took larger steps compared to the non-dominant limb), however, the effect of the exosuit was not strong enough to result in a significant post-effect between EP and BL. This is similar to recent results with a powered unilateral exosuit that produced a post-effect that was apparent in the mean but not significant [29]. The direction of this trend may explain the results seen in our simultaneous group (Exo-Sb). Participants from Exo-Sb walked with diminished positive step length asymmetry at EP and did not show the typical significant post-effect that we observed in Sb-only [47, 56]. In other words, these findings suggest the unilateral passive hip exosuit diminished the post-effect from split-belt walking. Participants in the Sb-only voluntarily expressed that they sensed the belts continued to move at different velocities at EP, despite the belts’ tied configuration. Participants in Exo-Sb did not openly express these same sensory observations, providing anecdotal evidence that the passive exosuit influenced typical split-belt gait characteristics and perceptions.

In line with previous literature, the interlimb symmetry results from this study confirm that passive exosuits have the potential to alter gait characteristics [4, 36]. The question that arises is: How did the passive exosuit aid the diminished step length asymmetry when the individual belt velocities returned to a tied configuration? When healthy participants walk on a treadmill with belts at different velocities, the leg on the fast belt takes shorter step lengths while the leg on the slow belt takes longer step lengths. After the belts return to a tied speed, the opposite occurs due to a temporary miscalibration of perception: the leg on the fast-belt switches to longer step lengths while the leg on the slow-belt shifts to shorter step lengths [47, 56]. Based on the trends in the adaptation conditions, it seems that the unilateral passive hip flexion resistance applied to the leg on the slow belt produced a similar effect as walking on a fast belt, thereby counteracting a portion of the effect of the split-belt adaptation.

The finding that it is possible to diminish induced step length asymmetry after-effects in healthy participants using a simple, lightweight design supports interest in future studies in patient populations with asymmetric gait. Recently, however, research has questioned whether diminishing gait asymmetry helps to improve gait in patients with hemiparesis. Musculoskeletal models, as well as experiments involving participants following stroke, found that acutely restoring step length symmetry did not improve the metabolic cost of transport and may even negatively impact dynamic balance in patient populations [61–64]. The authors argue that these results suggest maintaining a certain level of asymmetry leads to an optimal locomotive solution for stroke patients [61, 63, 65, 66]. However, it remains to be seen whether more long-term gait symmetry changes could result in benefits not seen with short-term changes [64]. The portability and low-cost of such devices would lend to longer training durations, rural clinical accessibility, and the opportunity to assess the long-term impact of symmetrical gait training on patient outcomes. Such long-term studies must assess how the passive exosuit may change gait, dynamic balance, cost of transport, and patient specific tuning compared to control groups that receive current best practice therapy. Another possible avenue for future research would involve assisting hip flexion on one limb while assisting hip extension on the other limb. This “asymmetrical” bilateral setup may produce significant post-effects that we did not find with the current Exo-only condition and it could lead to adaptations via a portable device that are similar to those of split-belt walking.

Although improving symmetry would seem to improve walking ability, it has been shown that after several weeks of training, gait asymmetry did not change [67]. Additionally, muscle activation patterns remained asymmetric [67]. It is possible that the stroke survivors discovered a way to optimize bilateral coordination without reducing asymmetry or that asymmetry may serve a functional purpose [68, 69]. Proving this may be the key to determining the mechanism of functional gait improvement and inform the design of rehabilitation paradigms that target mechanisms to accelerate recovery. Such solutions could come from investigating behavioral data from a dynamical systems perspective [70]. In that study, we showed that when an exoskeletal-assisted split-belt treadmill task was performed, there was a reduction in the duration and synchrony of coordination, in comparison to a non-exoskeletal-assisted control group. It is possible that exoskeletal-assisted gait, such as in this study, is characterized by reduced inter-limb coordination possibly for allowing gait patterns to be more explorative and flexible. Such flexibility may be the key to functional gait improvement rather than asymmetry reduction. This is important in rehabilitation of patients who suffer from coordination deficits. Whether this is also the case in the current study, is an avenue for future research.

This study had some methodological limitations. The participant recruitment for this experiment occurred through convenience sampling on a college campus. Despite the recruitment age range from 19 to 40 years old, the sample selected may not generalize to the entire population. Additionally, healthy young adults volunteered as participants for this investigation, and the findings herein may not relate to clinical populations. We tested the effect of the device when it was attached to the limb on the slow belt only. The impact of the device could change if we placed it on the limb on the fast belt. Depending on an individual’s size or movement, the positioning of the exosuit could have changed during the trial leading to errors when calculating the force of the band. We did not evaluate qualitative measures of the passive exosuit like comfort, fit, and aesthetics. Future investigations should evaluate these qualitative measures in patient populations to encourage adherence to a training protocol. Due to the scope of the present study, we have not yet processed a sufficiently large number of strides to investigate aspects such as variability and changes in relative phasing over time that inform how participants adapt to different attractors. Future research may benefit from applying analysis techniques from Dynamical Systems Theory to understand how the device impacts coordination patterns throughout the entirety of data collection.

## Conclusions

Our study found that wearing a unilateral passive hip exosuit while walking on a split-belt treadmill led to diminished step length asymmetry once the belts return to a tied configuration. The simple design suggests that practical, inexpensive devices can alter walking patterns in healthy individuals. The results from this study warrant future research investigating different band configuration setups, walking with the device for extended periods overground, as well as testing in patient populations walking with asymmetric stepping patterns. Determining the long-term potential of such devices could improve therapeutic interventions for patient populations and offers the opportunity for widespread accessible interventions.

## Electronic supplementary material

Below is the link to the electronic supplementary material.

Additional File 1: Determining Band Coefficient of Stiffness: Correlation graph used to determine the correlation of stiffness of the passive component on the exosuit.

Additional File 2: Within-Group Stance Time SI Comparisons: Individual group comparisons of stance time SI across analysis timepoints.

Additional File 3: Within-Group Swing Time SI Comparisons: Individual group comparisons of swing time SI across analysis timepoints.

Additional File 4: Within-Group Step Length SI Comparisons: Individual group comparisons of step length SI across analysis timepoints.

Additional File 5: Data Sheet - An excel sheet with the data used to run statistical analyses.

Additional File 6: Supplemental Tables - A word document containing all of the supplemental data tables.

## Data Availability

All data generated or analyzed during this study are included in this published article [and its supplementary information files].

## Declarations

### Authors’ information

KK currently holds a doctoral research assistantship position in the Mary Frances Early College of Education’s Department of Kinesiology’s Concussion Research Laboratory at the University of Georgia.

## Abbreviations

CNS: Central nervous System
CIMT: Constraint-Induced Movement Therapy
GRF: Ground Reaction Force
Exo-only: Exosuit Only Group
Sb-only: Split-belt Only Group
Exo-Sb: Simultaneous Group
ANOVA: Analysis of Variance
BL: Baseline
EA: Early Adaptation
LA: Late Adaptation
EP: Early Post-adaptation
LP: Late Post-adaptation
SI: Symmetry Index
