# Supplementary material for: Can a passive unilateral hip exosuit diminish walking asymmetry? A randomized trial
Source: J Neuroeng Rehabil. 2023 Jul 12;20:88. doi: 10.1186/s12984-023-01212-w (PMC10339586; doi:10.1186/s12984-023-01212-w)
Supplement: Supplementary file 1 — Additional File 1: Determining Band Coefficient of Stiffness: Correlation graph used to determine the correlation of stiffness of the passive component on the exosuit. [file 12984_2023_1212_MOESM1_ESM.pdf]

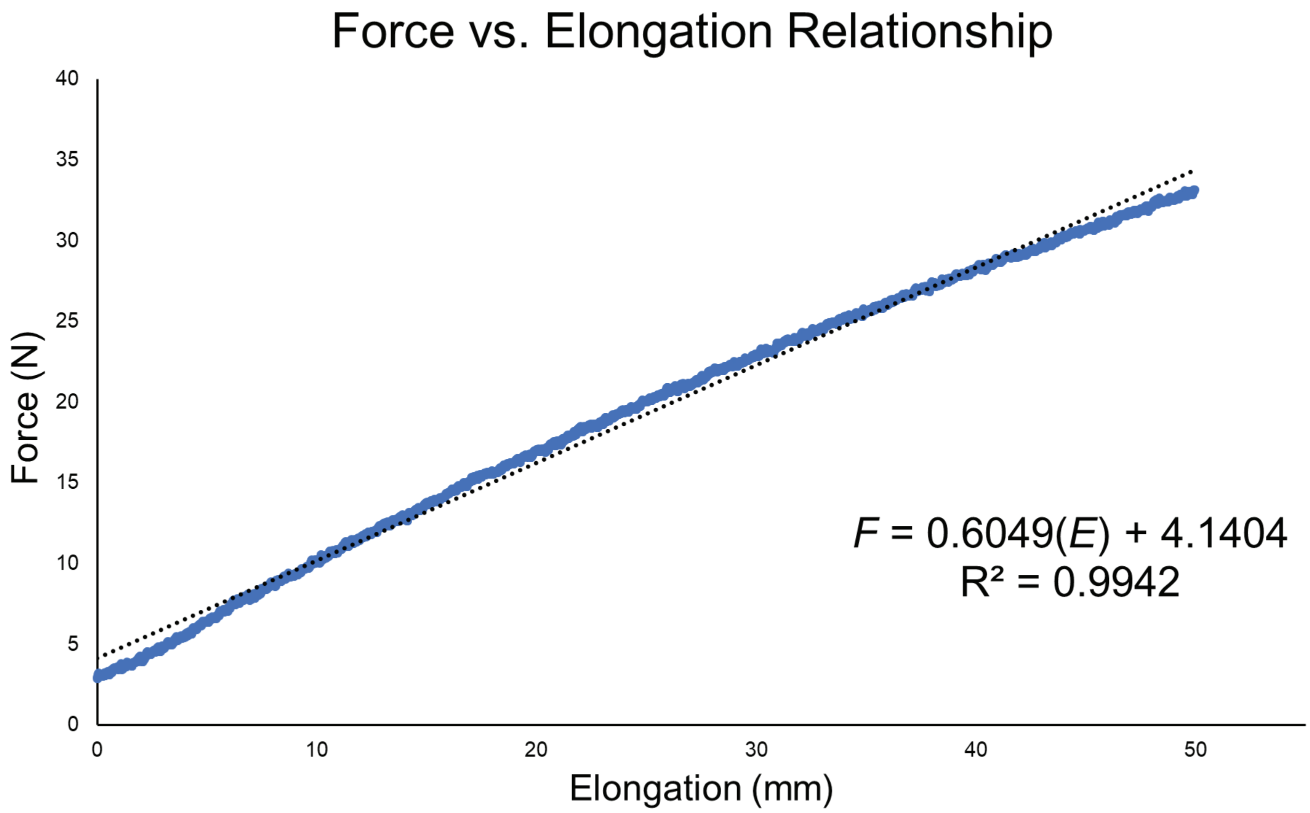

Additional File 1. Determining Band Coefficient of Stiffness. The force and elongation relationship for the FitSimplify heavy bands used as the elastic compenent of the passive hip exoskeleton. The line of best fit equation was found to have a significant postive correlation value (R^2 = 0.9942) and determined the coefficient of stiffness for the band (K_{el} = 0.6049).
