# Supplementary material for: Can a passive unilateral hip exosuit diminish walking asymmetry? A randomized trial
Source: J Neuroeng Rehabil. 2023 Jul 12;20:88. doi: 10.1186/s12984-023-01212-w (PMC10339586; doi:10.1186/s12984-023-01212-w)
Supplement: Supplementary file 3 — Additional File 3: Within-Group Swing Time SI Comparisons: Individual group comparisons of swing time SI across analysis timepoints. [file 12984_2023_1212_MOESM3_ESM.pdf]

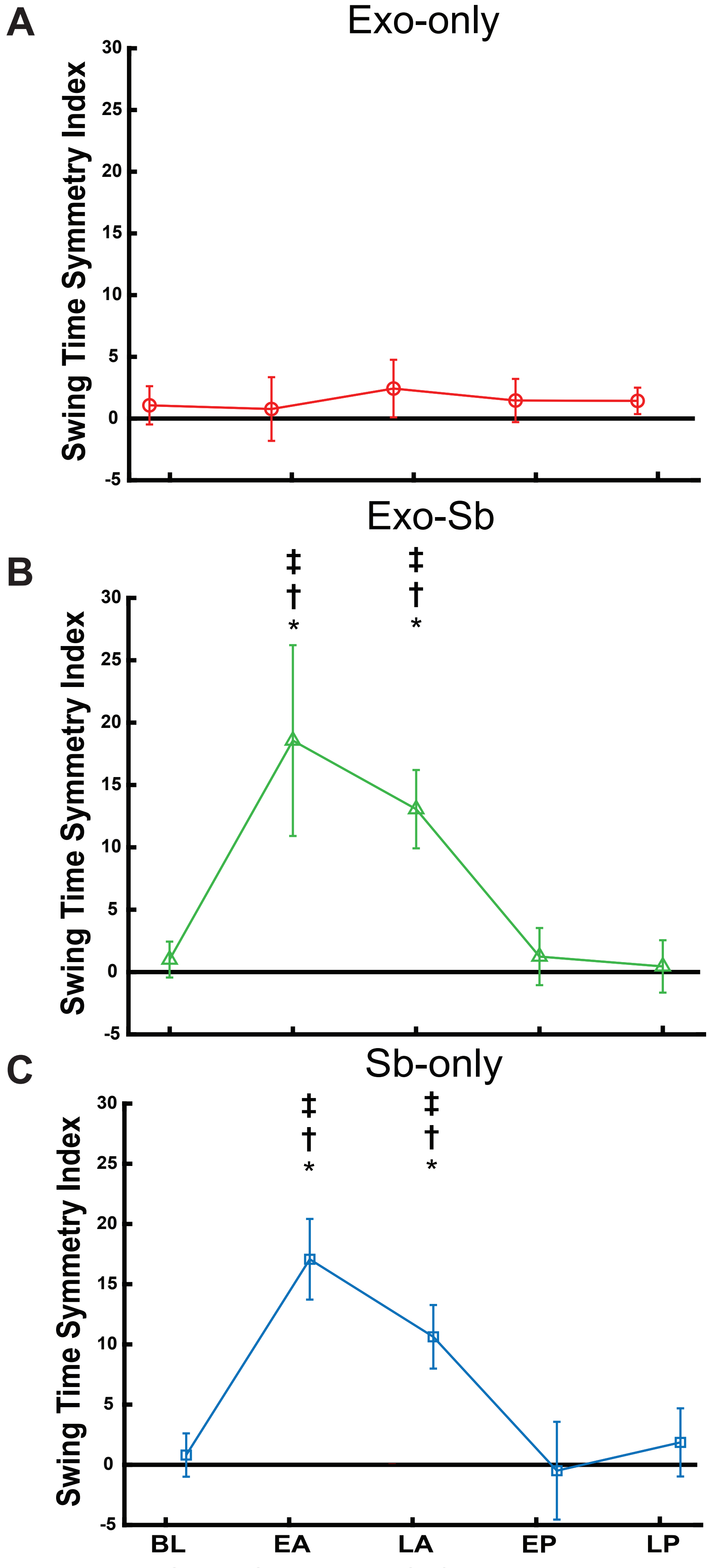

Additional File 3. Within-Group Swing Time SI Comparisons. Panel A, B, and C show swing time symmetry values for Exo-only, Exo-Sb, and Sb-only respectively. A "*" represents statistically significant differences from baseline (BL). A "†" represents statistically significant differences from early post-adaptation (EP) and a "‡" represented statistically significant differences from late post-adaptation (LP).
