# Supplementary material for: Can a passive unilateral hip exosuit diminish walking asymmetry? A randomized trial
Source: J Neuroeng Rehabil. 2023 Jul 12;20:88. doi: 10.1186/s12984-023-01212-w (PMC10339586; doi:10.1186/s12984-023-01212-w)
Supplement: Supplementary file 6 — Additional File 6: Supplemental Tables - A word document containing all of the supplemental data tables. [file 12984_2023_1212_MOESM6_ESM.pdf]

Table 1. *Stance Time SI Simple Effects ANOVA*

**Time**	**Effect**	**DFn**	**DFd**	**F**	**p**	**p_adj**	**η^2**
BL	Group	2	12	0.058	0.944	1	0.01
**EA**	**Group**	**2**	**12**	**13.80**	**< 0.001***	**0.004***	**0.697**
**LA**	**Group**	**2**	**12**	**13.10**	**< 0.001***	**0.005***	**0.686**
EP	Group	2	12	0.52	0.608	1	0.08
LP	Group	2	12	0.916	0.426	1	0.132

BL = Baseline, EA = Early Adaptation, LA = Late Adaptation, EP = Early Post-Adaptation, LP = Late Post-Adaptation, DFn = degrees of freedom numerator, DFd = degrees of freedom denominator, p_adj = adjusted p-value.

* Represents p values < 0.05

Bolded values highlight significant findings.

Table 2. *Swing Time SI Simple Effects ANOVA*

**Time**	**Effect**	**DFn**	**DFd**	**F**	**p**	**p_adj**	**η^2**
BL	Group	2	12	0.035	0.966	1	0.006
**EA**	**Group**	**2**	**12**	**19.10**	**< 0.001***	**< 0.001***	**0.761**
**LA**	**Group**	**2**	**12**	**20.90**	**< 0.001***	**< 0.001***	**0.777**
EP	Group	2	12	0.686	0.523	1	0.103
LP	Group	2	12	0.576	0.577	1	0.088

BL = Baseline, EA = Early Adaptation, LA = Late Adaptation, EP = Early Post-Adaptation, LP = Late Post-Adaptation, DFn = degrees of freedom numerator, DFd = degrees of freedom denominator, p_adj = adjusted p-value.

* Represents p values < 0.05

Bolded values highlight significant findings.

Table 3. *Sb-only Stance Time Paired Comparisons*

Contrast	Estimate	SE	df	t Ratio	p_adj	Effect Size	Lower CI	Upper CI
**BL-EA**	**7.941**	**1.29**	**16**	**6.166**	**0.0001***	**2.243**	**0.59**	**3.895**
**BL-LA**	**5.332**	**1.29**	**16**	**4.14**	**0.0077***	**1.506**	**0.258**	**2.754**
BL-EP	-0.562	1.29	16	-0.437	1	-0.159	-0.937	0.619
BL-LP	0.613	1.29	16	0.476	1	0.173	-0.606	0.952
EA-LA	-2.61	1.29	16	-2.026	0.5976	-0.737	-1.645	0.171
**EA-EP**	**-8.503**	**1.29**	**16**	**-6.602**	**0.0001***	**-2.401**	**-4.146**	**-0.657**
**EA-LP**	**-7.328**	**1.29**	**16**	**-5.69**	**0.0003***	**-2.07**	**-3.623**	**-0.516**
**LA-EP**	**-5.894**	**1.29**	**16**	**-4.576**	**0.0031***	**-1.664**	**-2.995**	**-0.334**
**LA-LP**	**-4.719**	**1.29**	**16**	**-3.664**	**0.021***	**-1.333**	**-2.494**	**-0.171**
EP-LP	1.175	1.29	16	0.912	1	0.332	-0.469	1.133

BL = Baseline, EA = Early Adaptation, LA = Late Adaptation, EP = Early Post-Adaptation, LP = Late Post-Adaptation, SE = standard error, df = degrees of freedom, p_adj = adjusted p-value.

* Represents p values < 0.05

Bolded values highlight significant findings.

Table 4. *Exo-Sb Stance Time Paired Comparisons*

Contrast	Estimate	SE	df	t Ratio	p_adj	Effect Size	Lower CI	Upper CI
**BL-EA**	**9.6068**	**1.24**	**16**	**7.744**	**< 0.0001***	**2.6617**	**0.687**	**4.636**
**BL-LA**	**6.7048**	**1.24**	**16**	**5.405**	**0.0006***	**1.8576**	**0.384**	**3.331**
BL-EP	0.0914	1.24	16	0.074	1	0.0253	-0.704	0.754
BL-LP	-0.4372	1.24	16	-0.352	1	-0.1211	-0.855	0.612
EA-LA	-2.902	1.24	16	-2.339	0.3262	-0.804	-1.72	0.112
**EA-EP**	**-9.5154**	**1.24**	**16**	**-7.67**	**< 0.0001***	**-2.6364**	**-4.595**	**-0.678**
**EA-LP**	**-10.044**	**1.24**	**16**	**-8.096**	**< 0.0001***	**-2.7828**	**-4.835**	**-0.73**
**LA-EP**	**-6.6134**	**1.24**	**16**	**-5.331**	**0.0007***	**-1.8323**	**-3.291**	**-0.374**
**LA-LP**	**-7.142**	**1.24**	**16**	**-5.757**	**0.0003***	**-1.9788**	**-3.526**	**-0.432**
EP-LP	-0.5287	1.24	16	-0.426	1	-0.1465	-0.882	0.589

BL = Baseline, EA = Early Adaptation, LA = Late Adaptation, EP = Early Post-Adaptation, LP = Late Post-Adaptation, SE = standard error, df = degrees of freedom, p_adj = adjusted p-value.

* Represents p values < 0.05

Bolded values highlight significant findings.

Table 5. *Sb-only Swing Time Paired Comparisons*

Contrast	Estimate	SE	df	t Ratio	p_adj	Effect Size	Lower CI	Upper CI
**BL-EA**	**-16.26**	**2.01**	**16**	**-8.09**	**< 0.0001***	**-3.123**	**-5.208**	**-1.038**
**BL-LA**	**-9.82**	**2.01**	**16**	**-4.889**	**0.0016***	**-1.887**	**-3.306**	**-0.469**
BL-EP	1.3	2.01	16	0.645	1	0.249	-0.583	1.082
BL-LP	-1.05	2.01	16	-0.522	1	-0.201	-1.029	0.626
EA-LA	6.43	2.01	16	3.201	0.0556	1.236	0.12	2.352
**EA-EP**	**17.55**	**2.01**	**16**	**8.735**	**< 0.0001***	**3.372**	**1.146**	**5.599**
**EA-LP**	**15.21**	**2.01**	**16**	**7.568**	**< 0.0001***	**2.922**	**0.95**	**4.893**
**LA-EP**	**11.12**	**2.01**	**16**	**5.534**	**0.0005***	**2.137**	**0.59**	**3.683**
**LA-LP**	**8.77**	**2.01**	**16**	**4.367**	**0.0048***	**1.686**	**0.366**	**3.005**
EP-LP	-2.53	2.01	16	-1.167	1	-0.451	-1.315	0.413

BL = Baseline, EA = Early Adaptation, LA = Late Adaptation, EP = Early Post-Adaptation, LP = Late Post-Adaptation, SE = standard error, df = degrees of freedom, p_adj = adjusted p-value.

* Represents p values < 0.05

Bolded values highlight significant findings.

Table 6. *Exo-Sb Swing Time Paired Comparisons*

Contrast	Estimate	SE	df	t Ratio	p_adj	Effect Size	Lower CI	Upper CI
**BL-EA**	**-17.561**	**2.18**	**16**	**-8.047**	**< 0.0001***	**-2.7165**	**-4.7536**	**-0.679**
**BL-LA**	**-12.053**	**2.18**	**16**	**-5.523**	**0.0005***	**-1.865**	**-3.3564**	**-0.373**
BL-EP	-0.233	2.18	16	-0.107	1	-0.0361	-0.7521	0.68
BL-LP	0.552	2.18	16	0.253	1	0.0853	-0.6328	0.803
EA-LA	5.508	2.18	16	2.524	0.2256	0.852	-0.0807	1.785
**EA-EP**	**17.327**	**2.18**	**16**	**7.94**	**< 0.0001***	**2.6803**	**0.6669**	**4.694**
**EA-LP**	**18.112**	**2.18**	**16**	**8.3**	**< 0.0001***	**2.8018**	**0.7084**	**4.895**
**LA-EP**	**11.819**	**2.18**	**16**	**5.416**	**0.0006***	**1.8284**	**0.3586**	**3.298**
**LA-LP**	**12.604**	**2.18**	**16**	**5.776**	**0.0003***	**1.9498**	**0.405**	**3.495**
EP-LP	0.785	2.18	16	0.36	1	0.1214	-0.5992	0.842

BL = Baseline, EA = Early Adaptation, LA = Late Adaptation, EP = Early Post-Adaptation, LP = Late Post-Adaptation, SE = standard error, df = degrees of freedom, p_adj = adjusted p-value.

* Represents p values < 0.05

Bolded values highlight significant findings.

Table 7. *Sb-only Step Length Paired Comparisons*

Contrast	Estimate	SE	df	t Ratio	p_adj	Effect Size	Lower CI	Upper CI
**BL-EA**	**13.02**	**1.21**	**16**	**10.774**	**< 0.0001***	**4.425**	**1.727**	**7.123**
BL-LA	2.19	1.21	16	1.811	0.8894	0.744	-0.227	1.714
**BL-EP**	**-9.47**	**1.21**	**16**	**-7.842**	**< 0.0001***	**-3.22**	**-5.273**	**-1.168**
BL-LP	-1.87	1.21	16	-1.547	1	-0.635	-1.58	0.309
**EA-LA**	**-10.83**	**1.21**	**16**	**-8.963**	**< 0.0001***	**-3.681**	**-5.977**	**-1.385**
**EA-EP**	**-22.49**	**1.21**	**16**	**-18.616**	**< 0.0001***	**-7.645**	**-12.143**	**-3.148**
**EA-LP**	**-14.89**	**1.21**	**16**	**-12.321**	**< 0.0001***	**-5.06**	**-8.107**	**-2.013**
**LA-EP**	**-11.66**	**1.21**	**16**	**-9.653**	**< 0.0001***	**-3.964**	**-6.412**	**-1.516**
**LA-LP**	**-4.06**	**1.21**	**16**	**-3.358**	**0.04***	**-1.379**	**-2.559**	**-0.199**
**EP-LP**	**7.61**	**1.21**	**16**	**6.295**	**0.0001***	**2.585**	**0.858**	**4.313**

BL = Baseline, EA = Early Adaptation, LA = Late Adaptation, EP = Early Post-Adaptation, LP = Late Post-Adaptation, SE = standard error, df = degrees of freedom, p_adj = adjusted p-value.

* Represents p values < 0.05

Bolded values highlight significant findings.

Table 8. *Exo-only Step Length Paired Comparisons*

**Contrast**	**Estimate**	**SE**	**df**	**t Ratio**	**p_adj**	**Effect Size**	**Lower CI**	**Upper CI**
**BL-EA**	**-2.186**	**0.649**	**16**	**-3.36**	**0.0392***	**-1.457**	**-2.6728**	**-0.2411**
BL-LA	-1.743	0.649	16	-2.685	0.1652	-1.162	-2.2784	-0.0455
BL-EP	1.497	0.649	16	2.305	0.3489	0.997	-0.0702	2.0651
BL-LP	-0.457	0.649	16	-0.705	1	-0.305	-1.2372	0.6274
EA-LA	0.443	0.649	16	0.682	1	0.295	-0.6364	1.2264
**EA-EP**	**3.683**	**0.649**	**16**	**5.672**	**0.0003***	**2.454**	**0.8269**	**4.0819**
EA-LP	1.729	0.649	16	2.663	0.1703	1.152	0.0387	2.2654
**LA-EP**	**3.24**	**0.649**	**16**	**4.991**	**0.0013***	**2.159**	**0.6626**	**3.6562**
LA-LP	1.286	0.649	16	1.981	0.6508	0.857	-0.1734	1.8875
EP-LP	-1.954	0.649	16	-3.01	0.0831	-1.302	-2.4644	-0.1403

BL = Baseline, EA = Early Adaptation, LA = Late Adaptation, EP = Early Post-Adaptation, LP = Late Post-Adaptation, SE = standard error, df = degrees of freedom, p_adj = adjusted p-value.

* Represents p values < 0.05

Bolded values highlight significant findings.

Table 9. *Exo-Sb Step Length Paired Comparisons*

Contrast	Estimate	SE	df	t Ratio	p_adj	Effect Size	Lower CI	Upper CI
**BL-EA**	**12.31**	**2.24**	**12**	**5.484**	**0.0014***	**3.877**	**1.565**	**6.19**
BL-LA	1.1	2.24	12	0.492	1	0.348	-1.2008	1.896
BL-EP	-7	2.24	12	-3.12	0.0885	-2.206	-4.033	-0.38
BL-LP	-1.65	2.24	12	-0.736	1	-0.52	-2.078	1.038
**EA-LA**	**-11.2**	**2.24**	**12**	**-4.992**	**0.0031***	**-3.53**	**-5.7294**	**-1.33**
**EA-EP**	**-19.31**	**2.24**	**12**	**-8.604**	**< 0.0001***	**-6.084**	**-9.1974**	**-2.97**
**EA-LP**	**-13.96**	**2.24**	**12**	**-6.219**	**0.0004***	**-4.397**	**-6.8872**	**-1.908**
**LA-EP**	**-8.11**	**2.24**	**12**	**-3.612**	**0.0357***	**-2.554**	**-4.4681**	**-0.64**
LA-LP	-2.75	2.24	12	-1.227	1	-0.868	-2.456	0.721
EP-LP	5.35	2.24	12	2.385	0.3446	1.686	-0.0272	3.4

BL = Baseline, EA = Early Adaptation, LA = Late Adaptation, EP = Early Post-Adaptation, LP = Late Post-Adaptation, SE = standard error, df = degrees of freedom, p_adj = adjusted p-value.

* Represents p values < 0.05

Bolded values highlight significant findings.

Table 10. Group Demographics

	Groups					
	Exo-only (n = 5)		*Sb-only (n = 5)*		*Exo-Sb (n = 5)*	
**Demographic**	**Mean**	**SD**	**Mean**	**SD**	**Mean**	**SD**
Age (year)	23.0	2.0	25.6	3.1	23.8	1.8
Weight (kg)	69.7	14.0	75.6	12.7	71.4	10.7
Height (m)	1.72	0.04	1.72	0.16	1.73	0.08
